# Identification and Unusual Properties of the Master Regulator FNR in the Extreme Acidophile *Acidithiobacillus ferrooxidans*

**DOI:** 10.3389/fmicb.2019.01642

**Published:** 2019-07-19

**Authors:** Héctor Osorio, Erin Mettert, Patricia Kiley, Mark Dopson, Eugenia Jedlicki, David S. Holmes

**Affiliations:** ^1^Center for Bioinformatics and Genome Biology, Fundación Ciencia y Vida, Santiago, Chile; ^2^Department of Biomolecular Chemistry, University of Wisconsin–Madison, Madison, WI, United States; ^3^Centre for Ecology and Evolution in Microbial Model Systems, Linnaeus University, Kalmar, Sweden; ^4^Universidad San Sebastian, Santiago, Chile; ^5^Centro de Genómica y Bioinformática, Facultad de Ciencias, Universidad Mayor, Santiago, Chile

**Keywords:** fumarate nitrate reductase, anaerobic regulation, transcriptional regulation, DNA binding, iron-sulfur cluster, biomining, microbial ecology

## Abstract

The ability to conserve energy in the presence or absence of oxygen provides a metabolic versatility that confers an advantage in natural ecosystems. The switch between alternative electron transport systems is controlled by the fumarate nitrate reduction transcription factor (FNR) that senses oxygen via an oxygen-sensitive [4Fe-4S]^2+^ iron-sulfur cluster. Under O_2_ limiting conditions, FNR plays a key role in allowing bacteria to transition from aerobic to anaerobic lifestyles. This is thought to occur via transcriptional activation of genes involved in anaerobic respiratory pathways and by repression of genes involved in aerobic energy production. The Proteobacterium *Acidithiobacillus ferrooxidans* is a model species for extremely acidophilic microorganisms that are capable of aerobic and anaerobic growth on elemental sulfur coupled to oxygen and ferric iron reduction, respectively. In this study, an FNR-like protein (FNR_AF_) was discovered in *At. ferrooxidans* that exhibits a primary amino acid sequence and major motifs and domains characteristic of the FNR family of proteins, including an effector binding domain with at least three of the four cysteines known to coordinate an [4Fe-4S]^2+^ center, a dimerization domain, and a DNA binding domain. Western blotting with antibodies against *Escherichia coli* FNR (FNR_EC_) recognized FNR_AF_. FNR_AF_ was able to drive expression from the FNR-responsive *E. coli* promoter P*narG*, suggesting that it is functionally active as an FNR-like protein. Upon air exposure, FNR_AF_ demonstrated an unusual lack of sensitivity to oxygen compared to the archetypal FNR_EC_. Comparison of the primary amino acid sequence of FNR_AF_ with that of other natural and mutated FNRs, including FNR_EC_, coupled with an analysis of the predicted tertiary structure of FNR_AF_ using the crystal structure of the related FNR from *Aliivibrio fisheri* as a template revealed a number of amino acid changes that could potentially stabilize FNR_AF_ in the presence of oxygen. These include a truncated N terminus and amino acid changes both around the putative Fe-S cluster coordinating cysteines and also in the dimer interface. Increased O_2_ stability could allow *At. ferrooxidans* to survive in environments with fluctuating O_2_ concentrations, providing an evolutionary advantage in natural, and engineered environments where oxygen gradients shape the bacterial community.

## Introduction

A central challenge in microbial ecology is to understand how microorganisms interact in complex communities, including how they respond to dynamically changing environments. Answers to this challenge are important for addressing issues such as the role of biogeochemical reactions in nutrient and energy cycling and in understanding ecosystem functioning in earth, ocean, and atmospheric environments (Widder et al., 2016). However, it is difficult to model such systems because of their complexity as well as that their experimental investigation in the field may require long time scales, measured in years to centuries, e.g., ecological succession.

Hyperacidic environments (pH < 3) provide an advantage over neutral milieu for addressing these issues as they generally exhibit low microbial diversity (Mendez-Garcia et al., 2015; Teng et al., 2017). This facilitates data collection, observation, and experimental exploration of ecological models over periods measured in weeks or months and simplifies model building of microbial interactions. One such environment is bioleaching heaps (termed “bioheaps”) that exploit acidophilic microorganisms’ metabolism (Bonnefoy and Holmes, 2012; Dopson and Johnson, 2012) to catalyze commercial metal recovery from sulfide minerals in many parts of the world (Brierley and Brierley, 2013; Vera et al., 2013). Bioheaps offer additional advantages for studying microbial community function and dynamics as they are subjected to dynamically changing conditions including levels of heavy metals, acidity, CO_2_, temperature, nutrients, and available redox couples for growth (Dopson et al., 2009; Valdés et al., 2010; Riekkola-Vanhanen, 2013; Tupikina et al., 2013; Dopson and Holmes, 2014). In addition, bioheap microbes are challenged by a gradient of O_2_ availability when thick biofilms are formed (Baker-Austin et al., 2010), due to limitations of O_2_ gas–liquid mass transfer that are exacerbated at higher temperatures (Petersen, 2010), and decreasing O_2_ concentrations in the center of the bioheap (Yin et al., 2011).

*Acidithiobacillus ferrooxidans* is a keystone bioheap species that is especially prevalent during early stage bioleaching (Demergasso et al., 2005; Remonsellez et al., 2009; Halinen et al., 2012). This is likely as it is able to grow at higher pH values than e.g., *Leptospirillum ferriphilum* (Dopson, 2016) and as it fixes carbon that aids in the subsequent growth of heterotrophic acidophilic species, as has been demonstrated during co-culture of autotrophic, and heterotrophic biomining species (Nancucheo and Johnson, 2010). *At. ferrooxidans* is an acidophilic, obligatory chemolithoautotrophic mesophile that gains its energy from the oxidation of ferrous iron, elemental sulfur, inorganic sulfur compounds, and hydrogen (Bonnefoy and Holmes, 2012; Dopson and Johnson, 2012; Hedrich and Johnson, 2013). *At. ferrooxidans* is a facultative anaerobe that grows under aerobic and anaerobic conditions and the main electron transport components couple the aerobic oxidation of iron and sulfur to the reduction of O_2_ (Quatrini et al., 2009) and the anaerobic oxidation of elemental sulfur to reduction of ferric iron (Pronk et al., 1991; Ohmura et al., 2002; Osorio et al., 2013). This switch from aerobic to anaerobic growth is expected to require a regulator of gene expression, which has not been studied in detail in *At. ferrooxidans*.

The transition from oxic, to hypoxic (low concentrations of O_2_), and finally anoxic environments may require gene regulation systems to respond to the varying O_2_ concentrations (Osorio et al., 2009). In addition, *At. ferrooxidans* requires iron homeostasis systems (such as for the ferric iron utilized as electron acceptor) as its concentration can reach 10^18^-fold higher than in pH neutral environments (Osorio et al., 2008a, b). Bacterial O_2_ sensing systems include the direct interaction of O_2_ with membrane sensors such as FixL and the cytoplasmic transcription factor FNR (Fumarate and Nitrate Reduction), along with redox responsive regulatory systems that include, but are not limited to, ArcBA and Rex (reviewed in (Green et al., 2009; Bueno et al., 2012; Mettert and Kiley, 2018). The FNR transcription factor is a member of the cyclic AMP (cAMP) receptor protein (Crp) superfamily and plays a major role in altering gene expression between oxic and anoxic conditions (Constantinidou et al., 2006). The *Escherichia coli* FNR protein (termed FNR_EC_) senses O_2_ via four cysteine residues that ligate an O_2_-sensitive [4Fe-4S]^2+^ iron-sulfur cluster in the N-terminal region and affects its regulatory function via the C-terminal helix-turn-helix (HTH) DNA binding domain (Fleischhacker and Kiley, 2011; Mettert and Kiley, 2018). In anoxic conditions, FNR is activated by Isc protein-dependent acquisition of the [4Fe-4S]^2+^ cluster that promotes dimerization. The dimer binds to target DNA sequences, and induces or represses transcription. As O_2_ levels increase, the FNR [4Fe-4S]^2+^ cluster is degraded and the protein is converted to a monomeric form, which is no longer active in gene regulation (Crack and Le Brun, 2018; Mettert and Kiley, 2018). *At. ferrooxidans* gene clusters predicted to be under the control of FNR are suggested to be involved in carbon and energy metabolism along with nitrogen fixation (Osorio et al., 2009). However, the predicted role of FNR and the mechanisms of adaption to changing O_2_ concentrations in *At. ferrooxidans* have not been experimentally tested.

Due to the importance of *At. ferrooxidans* as a model organism in natural and man-made acidic environments, it is important to understand the regulation of growth, adaptation, and extracellular electron transfer under anoxic and acidic conditions. Here, we characterized the *At. ferrooxidans* FNR master gene regulator and examined the effect of changing O_2_ concentrations on its Fe-S cluster ligand.

## Materials and Methods

### Bacterial Strains and Growth Conditions

*Acidithiobacillus ferrooxidans*^T^ ATCC 23270 was obtained from the American Type Culture Collection (Table 1). The strain was maintained in sterile 9K basal salts medium (sterilized by Tyndallization) adjusted to pH 3.5 with H_2_SO_4_ (Silverman and Lundgren, 1959) containing 0.5% (wt/vol) S^0^ and incubated under aerobic conditions at 30°C with shaking. Anaerobic (S^0^/Fe^3+^) cultures of *At. ferrooxidans* were grown in identical medium with the exception of the addition of 25 mM ferric iron (sterile filtered through a 0.22 μm filter) as electron acceptor and the pH was adjusted to 1.8. *At. ferrooxidans* was pre-grown in aerobic conditions before transferring the cells to an anaerobic jar using the Anaerocult A system (Merck).

**TABLE 1:** Bacterial strains and plasmids used in this study.

**Strain or plasmid**	**Genotype**	**References or sources**
*At. ferrooxidans*		
ATCC 23270	Wild type	
*Escherichia coli*		
JM109	endA1, recA1, gyrA96, thi, hsdR17 (rk–, mk+), relA1, supE44, Δ(lac-proAB), [F′ traD36, proAB, laqIqZΔM15]	Promega
BL21	F-, ompT, hsdSB (rB-mB-), gal, dcm, rne131, (DE3)	Invitrogen
PK22	hsdS, gaL λDE53, Δcrp-bs990, rpsL, Δfnr, zcj-3061::Tn10	Lazazzera et al., 1993
RZ7350	*lacZ Δ145, narG234::MudI1734*	Kiley and Reznikoff, 1991
RZ8480	*Δfnr, lacZ Δ145, narG234::MudI1734*	Lazazzera et al., 1993
Plasmids		
pET100/D-TOPO	Expression vector	Invitrogen
pET100/D-TOPO FNR_AF_	Expression vector, FNR_AF_ protein	This study
pKK223-3	Plasmid vector, Amp^r^	PL-Pharmacia
pKK223-3 *fnr*_AF_	*fnr*_AF_ coding region cloned into pKK223-3 vector with a tac promoter	This study
pET11A	Expresion vector, Amp^r^ with a T7 promoter	Novagen
pET11A *fnr*_AF_	*fnr*_AF_ coding region cloned into pET11A vector with a T7 promoter	This study

*Escherichia coli* strains (Table 1) were grown on a rotary shaker in sterile LB medium at 37°C. The following antibiotics were added as required: spectinomycin (Sp; 25 μg/mL), streptomycin (Sm; 25 μg/mL), ampicillin (Ap; 50 μg/mL), and tetracycline (Tc; 10 μg/mL). For β-galactosidase assays, *E. coli* strains were grown under aerobic or anaerobic conditions at 37°C in minimal medium M9 containing 0.1% glucose with the respective antibiotics. Anaerobic cultures were carried out in anaerobic jars (as described for *At. ferrooxidans*) on M9 minimal medium containing 0.1% (wt/vol) glucose.

### Ferrous Iron Production During Anaerobic Cultures

The formation of ferrous iron in anaerobic cultures was determined by titration with 2,2′-dypyridyl. Samples (1 mL) of culture medium were passed through a 0.2 μm membrane filter and 160 μL aliquots of the filtrate were added to 40 μL of 5 mM 2,2′-dypyridyl. The ferrous iron concentration was determined via a calibration curve of FeSO_4_ × 7H_2_O at an absorbance of 510 nm in a spectrophotometer. *At. ferrooxidans* growth was quantified by counting in a Petroff-Hausser chamber. The cells in aerobic and anaerobic cultures were quantified in triplicate cultures to construct the respective growth curves [data presented are means (*n* = 3) ± standard deviations].

### Bioinformatics

The amino acid sequence of FNR from *E. coli* K12 (FNR_EC_; accession number, WP_000916335) was used in a BlastP search against the genome of *At. ferrooxidans* ATCC 23270. A potential FNR candidate (FNR_AF_; locus tag, AFE_0270) was identified with 28% identity. Using FNR_AF_ in a reciprocal best Blast hit against the NCBI nr database recovered hits against the Crp-Fnr family of transcriptional regulators (domain architecture ID 11429533) from multiple organisms. A conserved domain analysis of FNR_AF_ was carried out (Marchler-Bauer et al., 2013). Multiple sequence alignments of FNR_AF,_ FNR_EC_ and FNR from *Aliivibrio fisheri* (FNR_AFI_, Q5E593) were carried out using Clustal Omega (Sievers and Higgins, 2018) and Swiss-Model (Waterhouse et al., 2018). Where there was a difference between the two alignment methods, the alignment by Swiss-Model was chosen.

Secondary structure analysis of FNR_AF_ was carried out using homology modeling by comparing the predicted protein with the FNR crystal structure from *A. fisheri* (PDB 5e44) with a sequence identity of 27.35% and full coverage of the complete protein (Volbeda et al., 2015). The homology model was constructed using Modeler V.9 (Eswar et al., 2008) and validated using the ADIT! Validation Server from PDB (Richardson et al., 2013).

### RNA Extraction and Real-Time PCR

Cells were harvested from *At. ferrooxidans* cultures (maximum 1 × 10^9^ total cells) by centrifugation at 12000 × *g* for 10 min at 4°C. The pellet was washed with 10 mM H_2_SO_4_ and then with TE buffer pH 8.0 and finally resuspended in 100 μL of TE pH 8.0. To this mixture, 10 μL RNAse-free lysis buffer (0.5 M TrisHCl, 20 mM EDTA, 10% SDS, pH 6.8) was added and mixed gently. The tubes were incubated at 100°C for 3 min and allowed to cool to room temperature. The previous steps were sufficient to guarantee the rupture of the cells without damaging the RNA. RNA was isolated using RNeasy Mini Kit (Qiagen®) and contaminant DNA removed using RNase free DNase I (Fermentas) according to the manufacturer’s recommendations. The RNA was resuspended in five volumes of RNA*later* solution (Qiagen) and subsequently frozen at −80°C until use. RNA samples were reverse-transcribed using Revertaid M-MuLV (Fermentas) and specific oligonucleotides (Table 2) according to the manufacturer’s recommendations and 0.5 μg of total RNA for each reaction. The real-time PCR reactions were performed using an iCycler thermal cycler (Bio-Rad) and the KAPA SYBR FAST qPCR kit (KAPABIOSYSTEMS). The 20 μL PCR reactions contained 2 μL of a 1:100 diluted cDNA sample, 200 nM of each primer (Table 2), and 1 × KAPA SYBR FAST qPCR Master Mix. The reference dye ROX was included at a final concentration of 5 nM. The cycling protocol was as follows: initial denaturation for 10 min at 95°C followed by 40 cycles of 30 s each at 95°C, 56°C, and 72°C. Fluorescence was measured after the extension phase at 72°C and specific amplification was confirmed by a single peak in the melting curve. For each experimental condition, total RNA was extracted from replicate *At. ferrooxidans* cultures and the real-time PCR reactions were performed in triplicate and thus, the data sets consist of six values per gene. Relative expression levels of *At. ferrooxidans fnr* (amplified with qPCR *fnr* FF and qPCR *fnr* REV primers; Table 2) were normalized with the expression of the stable reference gene *rpoC* (amplified with qPCR *rpo*C FF and qPCR *rpo*C REV primers; Table 2). The *rpoC* gene has previously been demonstrated to be expressed at a constant level and is a valid choice as a reference (Nieto et al., 2009). Stationary phase genomic DNA (10-fold dilutions ranging from 10 ng to 1 pg) was used to generate a five-point standard curve for every gene by using the Cycle Threshold (Ct) value vs. the logarithm of each dilution factor. Reaction efficiency {E = [10(−1/slope)]^–1^} for every gene was derived from the slope of the corresponding standard curves. A one-way Anova (multiple comparison analysis) or a two way ANOVA test swere carried out to test the statistical significance of gene expression results (McDonald, 2009) using the Graphpad Prism software^1^.

**TABLE 2:** Oligonucleotides used in this study.

**Name**	**Sequence (5′–3′)**	**Function**
pKK FF *fnr*-*Eco*RI	ATCGATGAATTCATGACTGCCAGGCACTCCG	Cloning
pKK REV *fnr*-*Eco*RI	ATCGATGAATTCTCAGGCGCGGGTGCC	Cloning
*fnr* FF-NdeI	CATATGACTGCCAGGCACTCC	Cloning
*fnr* REV-NdeI	CATATGGTCTGCATTGACAATTATCAA	Cloning
qPCR *fnr* FF	AAGCTGGTCAAGAGTCTGCCCAAT	RT-qPCR
qPCR *fnr* REV	TGCCGGTCAAGGTAATGGCACTAT	RT-qPCR
qPCR *rpo*C FF	AATGCGGTGTTGAGGTAACC	RT-qPCR
qPCR *rpo*C REV	AGGTACTGGTCTTCGGTAAG	RT-qPCR

### Cloning Procedures

*Acidithiobacillus ferrooxidans* genomic DNA was prepared using the Wizard^®^ Genomic DNA Purification Kit (Promega Corp.). Plasmid DNA was prepared from *E. coli* JM109 cultures with the QIAprep^®^ Spin MiniPrep Kit (Qiagen). PCR products for cloning were amplified using oligonucleotides in Table 2 and purified from agarose gels with the SpinPrepTM Gel DNA Kit (Novagen).

To carry out β-galactosidase assays, the coding region of the *At. ferrooxidans fnr* gene (termed *fnr*_AF_) was amplified with primers containing embedded *Eco*RI restriction sites: pKK FF *fnr*-*Eco*RI and pKK REV *fnr*-*Eco*RI (Table 2). Cloning and transformation was carried out using standard techniques as described by Miller (1972). The amplification product was cloned into the multiple cloning site of pKK223-3 carrying the P*tac* promoter (Pharmacia Biotech), generating pKK223-3 *fnr*_AF_. pKK223-3 *fnr*_AF_ was transformed into *E. coli* strain RZ8480 which is Δ*fnr* and contains *lac*Z under control of the P*narG* promoter (Lazazzera et al., 1993). Finally, the *fnr*_AF_ coding region was cloned from PKK223-3 FNR_AF_ into the pET100/D-TOPO expression vector to generate pET100/D-TOPO FNR_AF_ that was subsequently used as a control in the Western blot analysis.

For the construction of the plasmid pET11A *fnr*_AF,_the *At. ferrooxidans fnr* gene coding region was amplified with primers containing embedded *Nde*I restriction sites *fnr* FF-*Nde*I and *fnr* REV-NdeI (Table 2). The product was cloned into pET11A (Novagen), generating plasmid pET11A *fnr*_AF_ (Table 2). The construct was then transformed into *E. coli* strain PK22 (Lazazzera et al., 1993) for FNR_AF_ purification experiments. *E. coli* strain PK22 was used because it lacks both *FNR*_EC_ and the structurally related CRP (cAMP-activated global transcriptional regulator) that could potentially contaminate the preparation of FNR_AF_ (Lazazzera et al., 1993).

### β-Galactosidase Assays

*Escherichia coli* was pre-grown aerobically overnight in M9-glucose medium (Sambrook et al., 1989). The medium (10 mL) was inoculated with 1% (vol/vol) seed culture and incubated in anaerobic jars until the cell density reached an optical density at 600 nm (OD_600_) of 0.8. The cultures had not yet achieved stationary phase since the cell mass increased at least twofold with further incubation. β-galactosidase assays were performed as described by (Miller, 1972) using chloroform and 0.1% sodium dodecyl sulfate to permeabilize the cells. All β-galactosidase assay results are the average of triplicate samples for each strain ± standard deviations.

### Purification of FNR_AF_

FNR-like protein was purified from strain PK22 carrying pET11A-*fnr*AF using the anaerobic protocol developed for FNR_EC_. The cells were grown aerobically in 4 L of M9 minimal medium plus 0.2% (wt/vol) glucose and ampicillin at 37°C to an OD_600_ ∼0.3, and IPTG was added to a final concentration of 400 μM for 1 h to induce FNR biosynthesis. After induction, cells were sparged overnight at 4°C with argon to remove the presence of O_2_. All subsequent steps in FNR purification were carried out under anaerobic conditions in a Coy anaerobic chamber with an atmosphere of 90% N_2_ and 10% H_2_ or in sealed tubes. The cells were harvested by centrifugation at 7,900 *g* for 15 min at 4°C and concentrated 200-fold in buffer A [50 mM potassium phosphate (pH 6.8), 0.1 mM EDTA, 0.1 M KC1, 10% glycerol, 1 mM dithiothreitol (DTT), and 0.1 mM phenylmethanesulfonylfloride (PMSF)], and passed once through a French press at 20,000 psi. The extracts were centrifuged at 139,000 *g* for 1 h to remove the membrane fraction. Cell extracts were passed over a 5 mL Bio-Rex 70 cation-exchange column (BioRad Laboratories) at a flow rate of 0.17 mL/min and eluted with a 70 mL linear gradient of 0.1 to 1 M KC1 in buffer A. Fractions containing a green color were pooled and diluted 1:4 with buffer C [50 mM phosphate (pH 6.8) plus 10% (vol/vol) glycerol] and loaded onto a 1 mL BioRex-70 gravity column, washed with 2 column volumes of buffer A, and eluted with 1 column volume of buffer B [50 mM phosphate (pH 6.8), 10% (vol/vol) glycerol, and 1 M KCl]. The purity of the FNR protein preparations was estimated from Coomassie-stained SDS-polyacrylamide gels. The protein concentration was estimated by a Bradford assay using the Coomassie Plus Protein Assay Reagent (Pierce).

Purification of His-tagged FNR_AF_ was carried out by first transforming pET100/D-TOPO FNR_AF_ into *E. coli* BL21 cells. Induction of FNR_AF_ was carried out in cell cultures grown to an OD_600_ of 0.8 in LB supplemented with amp (100 μg/ml) by adding 1 mM IPTG to the culture medium for 1 h. The overexpressed FNR_AF_ protein contained in the soluble extracts was purified by nickel-charged agarose resins (BIO-RAD) using 1M imidazole.

### Western Blotting

Aliquots of total protein extract and purified FNR protein (approximately 10 μM of protein) were separated by SDS-PAGE with either 15 or 18% acrylamide (total acrylamide/bisacrylamide) and transferred onto nitrocellulose filters by standard methods with a Bio-Rad blotting apparatus.

The blotted proteins were subsequently screened using a polyclonal rabbit anti-FNR serum generated against FNR_EC._ Filters were blocked overnight in blocking solution [5% skimmed milk, 0.05% Triton X-100, and Tris-buffered saline (TBS)] at 4°C with agitation, incubated for 1 h with a 1:500 dilution of the primary antibody in TBS/Tween 20 (0.05%) and further incubated in a 1:15,000 dilution of peroxidase-conjugated anti-rabbit immunoglobulin in TBS/Tween 20 (0.05%) for another hour. Immunoreactive proteins were detected using the Supersignal West Pico chemiluminescent substrate (Pierce). Pre-stained broad-range molecular mass protein standards from Bio-Rad were used. Protein concentrations were determined with Bio-Rad Protein Assay using BSA as standard.

### UV-Visible Spectroscopy

To measure the absorbance of the FNR_AF_, 1 mL of the protein stored under anaerobic conditions was used at a concentration of 10 μM in 50 mM phosphate buffer pH 6.8 containing 0.4 M KCl and its absorbance was recorded between 200 to 700 nm in a Lambda 25 UV/Vis spectrophotometer (PerkinElmer). The impact of O_2_ on the spectral characteristics of Fnr_AF_ was evaluated by exposure of the sample to air for 0, 30, 90, 120, 150, 180, and 210 min and the absorbance spectra between 200 and 700 nm was recorded. As a control, the absorbance of the protein under anaerobic conditions was measured over the same time period in order to rule out other environmental factors causing a change in the protein’s spectral properties.

Iron determinations in FNR_AF_ were performed by the TPTZ method which forms a deep blue-purple color with ferrous iron that is spectrophotometrically measured at 562 nm as previously described (Yan and Kiley, 2009).

## Results and Discussion

### Anaerobic Growth of *At. ferrooxidans* ATCC 23270

**FIGURE 1:**
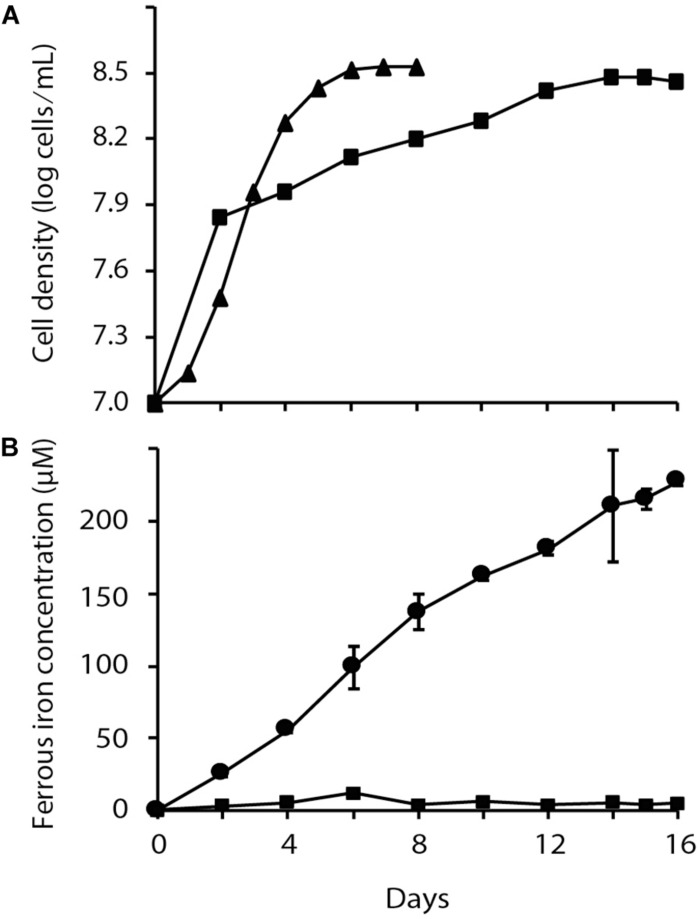
Chemolithoautotrophic growth of *Acidithiobacillus ferrooxidans* via aerobic and anaerobic respiration with S^0^ as the electron donor. **(A)** Time dependent changes in cell density of aerobically (▲) and anaerobically (■) respiring cultures and **(B)** Fe^2+^ formation in aerobic (■) and anaerobic (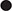) cultures. Data points are biological triplicates ± SD.

A comparison of *At. ferrooxidans* growth in oxic and anoxic conditions was performed in which elemental sulfur oxidation was coupled, respectively to reduction of O_2_ or Fe^3+^ as final electron acceptors. Despite the fact that both cultures reached similar levels of cell density, a reduced growth rate was observed when *At. ferrooxidans* used Fe^3+^ anaerobically instead of O_2_ as terminal electron acceptor (Figure 1). During anaerobic reduction of Fe^3+^, the amount of Fe^2+^ rose to a maximum of 228 ± 4 μM at stationary phase in the presence of *At. ferrooxidans* compared to 4 ± 4 μM Fe^2+^ in the un-inoculated control. These growth curves confirm and extend earlier observations (Osorio et al., 2013).

### Primary and Secondary Structure Analysis of FNR_AF_

The amino acid sequence of FNR_EC_ from *E. coli* K12 (accession number: WP_000916335) was used in a BlastP search against the genome of *At. ferrooxidans* ATCC 23270. A potential FNR_AF_ candidate (AFE_0270) was identified with 28% identity. Using FNR_AF_ in a reciprocal best Blast hit against the NCBI nr database recovered hits against the Crp-Fnr family of transcriptional regulators (domain architecture ID 11429533) from multiple organisms.

**FIGURE 2:**
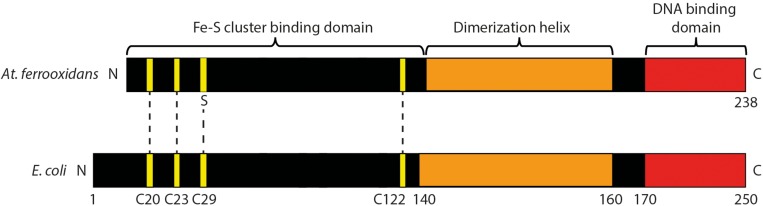
Cartoon of predicted domain structure of FNR_AF_ compared to FNR_EC_. The four cysteines that coordinate the [4Fe-4S]^2+^ cluster at positions 20, 23, 29, and 122 in FNR_EC_ (Mettert and Kiley, 2007) are highlighted in yellow. Three of these cysteines are conserved in FNR_AF_.

A conserved domain analysis of FNR_AF_ (Marchler-Bauer et al., 2013) and a comparison of its primary amino acid sequence with FNR_EC_ [as reviewed in (Crack and Le Brun, 2018; Mettert and Kiley, 2018)] showed that it contained the following motifs characteristic of an FNR-like protein^2^ : (i) three of the four cysteines (positions 20, 23, and 122, using a numbering system based on the FNR_EC_ sequence) potentially forming part of an Fe-S cluster binding domain involved in coordinating an [4Fe-4S]^2+^ center; (ii) a dimerization helix; and (iii) a DNA binding domain (Figure 2).

### FNR_AF_ Can Drive Expression From the FNR-Responsive *E. coli* Promoter P*narG*

**FIGURE 3:**
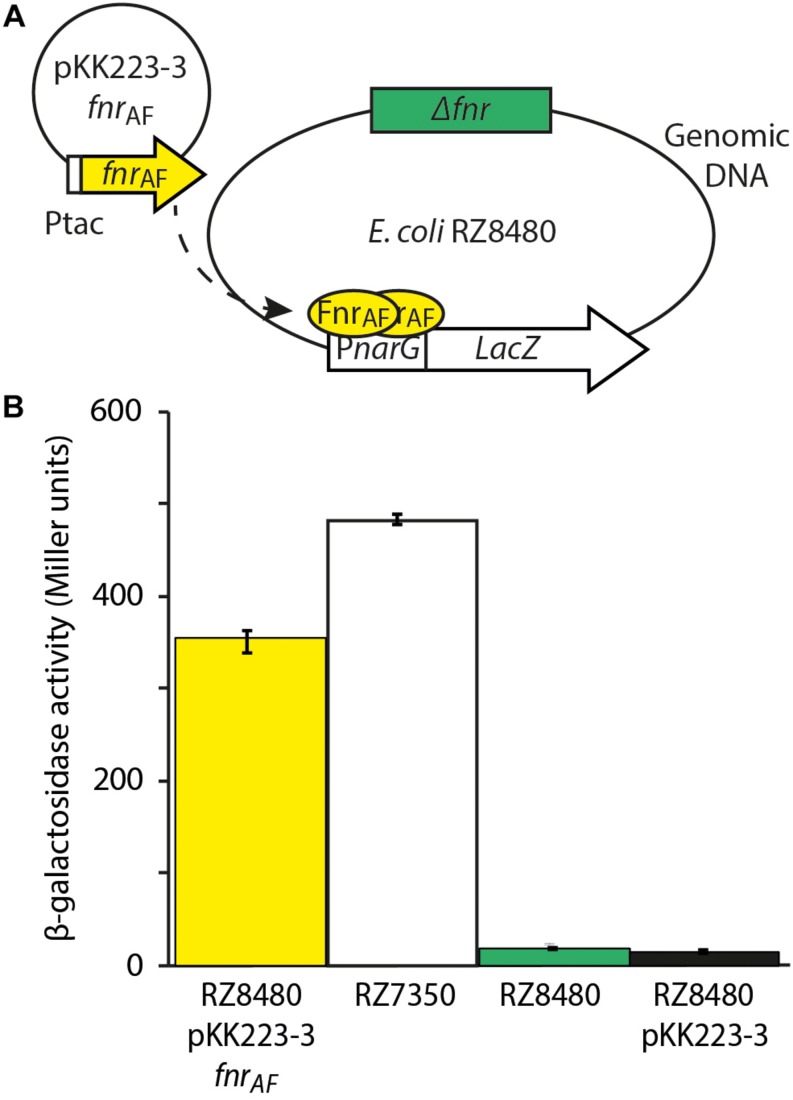
Experimental design and results of FNR_AF_ activity from the *Escherichia coli* promoter P*nar*G. **(A)** Experimental design to test the effect of FNR_AF_ (encoded on plasmid pKK223-3 *fnr*_AF_) on *in vivo* expression of P*nar*G in *E. coli Δfnr*. **(B)** Data are reported as β-galactosidase activities (*n* = 3 ± SD, one way Anova *p* < 0.05) in strains RZ8480 *A. f.* [*E. coli fnr* (−) *fnr A,f.* (+)] in plasmid pKK223-3, PK7350 [*E. coli fnr* (+)], RZ8480 [*E. coli fnr* (−)], and RZ8480 PKK [*E. coli fnr* (–)] with pKK223-3 plasmid. The dotted arrow indicates the expression of FNR_AF_ from *fnr*_AF_, its dimerization and binding to P*nar*G.

Although bioinformatic analyses strongly support the contention that FNR_AF_ is an FNR-like protein, we investigated whether FNR_AF_ could complement a mutant strain that lacked *fnr*, providing evidence for its function. It is difficult to generate a Δ*fnr* mutant of *At. ferrooxidans* as the organism is challenging to manipulate genetically (Inaba et al., 2018) as reviewed in (Gumulya et al., 2018). Therefore, we chose to complement an *E. coli* strain RZ8480 lacking *fnr* (Δ*fnr*). To accomplish this, a plasmid pKK223-3 *fnr*_AF_ was constructed containing the predicted *fnr*_AF_ coding sequence fused to the IPTG inducible promoter Ptac, and was transformed into RZ8480 (Δ*fnr*, P*narG–lacZ*). This strain contains *lac*Z, under the control of the FNR inducible P*narG* promoter (Figure 3A). Therefore, when a functional FNR is cloned and expressed in *E. coli* strain RZ8480, it can induce the expression of *lac*Z, giving rise to a measurable β-galactosidase activity.

*Escherichia coli* strain RZ8480 transformed with pKK223-3 *fnr*_AF_ was grown anaerobically until mid-log and β-galactosidase activity was measured after induction with 0.5 mM IPTG for 1 hr (Figure 3B). This activity was compared to that of *E. coli* strain RZ7350 that contains a native *fnr* and the P*narG-lacZ* allele. We observed that β-galactosidase was expressed in the recombinant strain harboring FNR_AF_, indicating FNR_AF_ is able to drive expression from the P*narG* promoter. However, expression from P*narG* is about 70% of that produced by FNR_EC_. One-way ANOVA (multiple comparisons analysis) was used to test the statistical significance yielding *p* < 0.05. Possible explanations for the observed decrease in expression are that there are important amino acid and/or structural differences between the respective FNRs or the architecture of the respective FNR binding sites are different. Alternatively, since P*tac* is a strong promoter, a certain amount of expressed FNR_AF_ could be present in the cell in a non-soluble form potentially accounting, at least in part, for the lower activity.

As expected, little β-galactosidase activity was detected in the *E. coli* strain RZ8480 transformed with the vector only control (pKK223-3 lacking *fnr*_AF_). Thus, *fnr*_AF_ is driving the expression of β-galactosidase and hence is capable of complementing Δ*fnr*_EC_.

The observation that *fnr*_AF_ can regulate expression from the *E. coli* P*narG* promoter provides evidence that it could potentially be involved in the regulation of anaerobic metabolism in *At. ferrooxidans* as has been observed in a number of organisms. However, as yet, there is no experimental evidence to test this hypothesis because of the difficulties involved in genetic manipulation of this organism.

### Transcription Levels of *At. ferrooxidans fnr* in Different Growth Conditions

**FIGURE 4:**
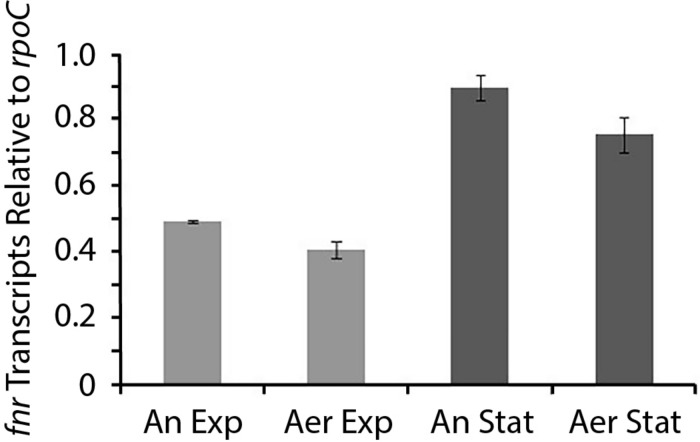
Level of *fnr*_AF_ transcripts in aerobic and anaerobic conditions. RT-qPCR data are expressed as relative values normalized to the housekeeping gene *rpoC* and presented as the average of triplicate samples ± the standard deviation, *two-way Anova p < 0.05*. An Exp, anaerobic exponential; Aer Exp, aerobic exponential; An Stat, anaerobic stationary; and Aer Stat, aerobic stationary.

Having demonstrated that FNR_AF_ is functional in a surrogate host, we wished to evaluate whether it was expressed in cell cultures of *At. ferrooxidans* and if so, under what conditions. Whole cell RNA was prepared from cells in four culture conditions: (i) anaerobic exponential growth; (ii) aerobic exponential growth; (iii) anaerobic stationary phase; and (iv) aerobic stationary phase and was quantified by RT-qPCR using the housekeeping gene *rpo*C mRNA as an internal standard (Figure 4). In anaerobic stationary phase, the number of RNA transcripts of FNR_AF_ exceeded that observed in aerobic conditions with statistical support (two-way ANOVA, multiple t unpaired test, *p* < 0.05). Also, the number of RNA transcripts in the stationary phase in both anaerobic and aerobic conditions exceeded (*p* < 0.05) those detected in the equivalent exponential phase. These results suggest the existence of a mechanism for regulating the level of *fnr*_AF_ transcripts depending on the growth phase and the presence or absence of O_2_. Using known transcription factor binding sites of *fnr*_AF_ as models (Osorio et al., 2009), no FNR-type binding sites could be detected bioinformatically upstream of *fnr*_AF_, suggesting that it is not auto-regulated.

### Purification and Biochemical Characterization of FNR_AF_

**FIGURE 5:**
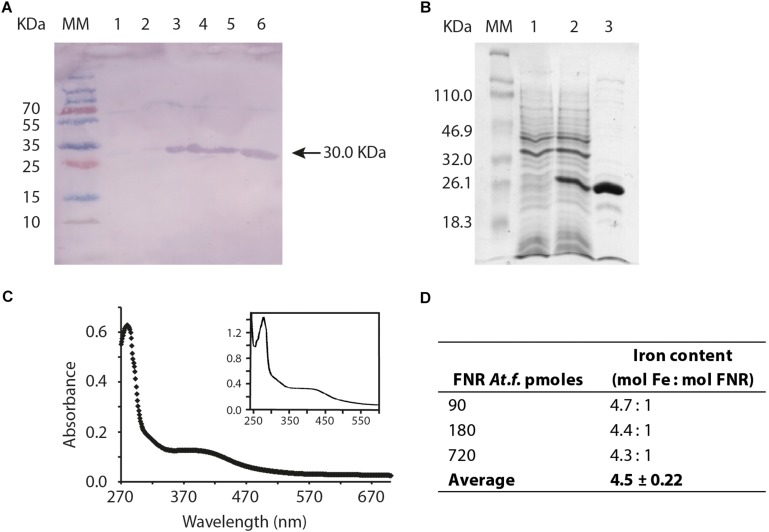
Biochemical validation and properties of FNR_AF_. **(A)** Western blot, antibodies prepared against FNR_EC_ were used to detect FNR_AF_, which was overproduced from plasmid pET100/D-TOPO *fnr*_AF_ cloned into *E. coli* strain BL21. The gel shows the molecular mass marker (MM), crude cell free extract (approximately 10 μg of total protein) prepared prior to induction of *fnr*_AF_ expression with IPTG (lane 1), 10 μg crude cell free extract 1 h after induction of *fnr*_AF_ expression with IPTG (lane 2), and elution’s 1 to 4 of 10 μg purified FNR_AF_ protein after elution from the nickel column (lanes 3–6). **(B)** Overproduction and purification of FNR_AF_ from the pET11A *fnr*_AF_ plasmid as shown by SDS-PAGE analysis. Gel lane 1 shows crude cell free extract (approximately 10 μg of total protein) prepared prior to induction of *fnr*_AF_ expression with IPTG; lane 2, 10 μg of crude cell free extract 1 h after induction of *fnr*_AF_ expression with IPTG; and lane 3, 10 μg FNR_AF_ after the second cationic interchange chromatographic column. M, molecular mass marker (*M*r are indicated). **(C)** Ultraviolet/visible absorption spectrum of a representative result of biological duplicates for purified FNR_AF_ from anaerobically grown *At. ferrooxidans* with an inset of the ultraviolet/visible spectrum of anaerobically purified FNR_*EC*_ taken from Lazazzera et al. (1993). **(D)** Calculation of the FNR_AF_ iron content by spectrophotometric assay.

Antibodies prepared against FNR_EC_ were able to react with FNR_AF_ prepared from plasmid pET100/D-TOPO *fnr*_AF_ cloned into *E. coli* strain BL21 consistent with the observation that the two FNRs have similar structural regions that the antibody recognizes (Figure 5A). To determine if FNR_AF_ contains an O_2_-sensitive metal cofactor, it was purified under anaerobic conditions (Figure 5B). The FNR_AF_ enriched fractions had a brownish color suggesting the presence of a light absorbing cofactor associated with the protein (data not shown). The ultraviolet/visible spectrum of the protein, recorded under anoxic conditions (Figure 5C), showed the expected protein absorption maximum at 280 nm and a broad absorbance centered around 420 nm, consistent with a Fe-S cluster containing protein (Khoroshilova et al., 1995). In order to identify the type of Fe-S cluster coordinated by FNR_AF_, we measured the iron content of the purified FNR_AF._ We found approximately 4.5 mol iron per monomer of mol FNR_AF_, which is highly suggestive of a [4Fe-4S]^2+^ cluster per monomer of protein (Figure 5D).

### FNR_AF_ Reacts More Slowly With O_2_ *in vitro* Than FNR_EC_

**FIGURE 6:**
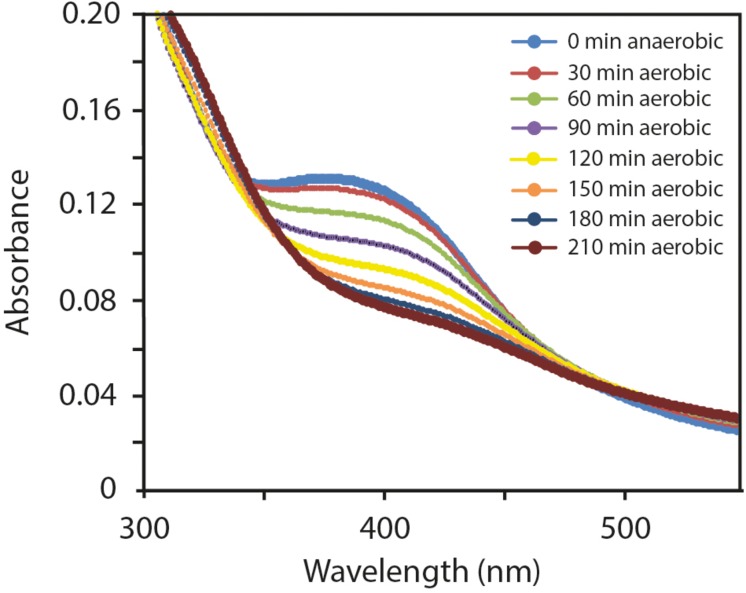
Effect of O_2_ exposure on the ultraviolet/visible spectrum of anoxically purified FNR_AF_ (10 μM). Changes are shown occurring in the 300–550 nm spectral region after 0, 30, 60, 90, 120, 150, 180, and 210 minutes exposure to O_2_.

The ability to sense and adapt to changes in O_2_ concentration is critical for the regulatory function of FNR proteins. The ability of FNR to function as a transcription factor depends on the integrity of the [4Fe-4S]^2+^ cluster, which promotes a conformation amenable for dimerization, site-specific DNA binding, and transcriptional regulation [reviewed in (Crack and Le Brun, 2018; Mettert and Kiley, 2018)]. The O_2_ sensitivity of FNR is mediated by the [4Fe-4S]^2+^ cluster whereby in the presence of O_2_, the [4Fe-4S]^2+^ cluster is converted to [2Fe-2S]^2+^ both *in vitro* and *in vivo*. The [2Fe-2S]^2+^ form of FNR is monomeric in solution and is inactive for DNA binding and transcriptional regulation (Jordan et al., 1997; Khoroshilova et al., 1997; Popescu et al., 1998). To test whether FNR_AF_ is O_2_ sensitive, the UV-visible spectrum of anaerobically purified FNR_AF_ was recorded after it was exposed to air. A progressive decrease in absorbance, was observed consistent with the degradation of the [4Fe-4S]^2+^ by O_2_ and with complete degradation occurring by 210 min (Figure 6). The cluster decay was much slower than that observed for wild type FNR_EC_ (Crack et al., 2014) and other naturally O_2_-stable FNRs from *Neisseria meningitidis* (Edwards et al., 2010), *Pseudomonas putida* [FNR PP_3233; (Ibrahim et al., 2015)], and *Paracoccus denitrificans* (Crack et al., 2016). Furthermore, the appearance of a [2Fe-2S]^2+^ cluster product was not readily observed as found previously with *E. coli* FNR.

### Primary Amino Acid Sequence Differences Between FNR_AF_ and FNR_EC_ Discussed in Light of the Three-Dimensional Crystal Structure of FNR From *Aliivibrio fisheri*

Note that in the following results, all amino acid locations in FNR_AF_ are given based on the numbering system of FNR_EC_ in order to expedite comparisons in the text between the two sequences which have different lengths.

**FIGURE 7:**
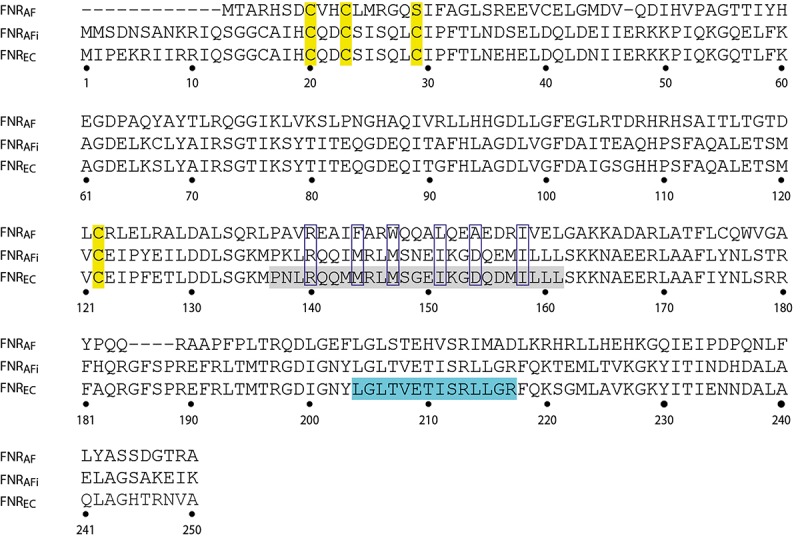
Comparison of the amino acid sequence of FNR_AF_ (AFE_0270) with that of FNR_EC_ (WP_000916335). The sequence of FNR from *Aliivibrio fisheri* (FNR_AFi_; Q5E593) is included for comparison. The numbers correspond to the amino acid sequence of FNR_EC_. In the sequence of FNR_EC_, the four cysteines coordinating the [4Fe-4S]^2+^ center are highlighted in yellow. FNR_EC_ sequences corresponding to the dimerization helix and the DNA binding HTH motif are highlighted in gray and turquoise, respectively. Clear boxes indicate additional amino acids discussed in the text.

**FIGURE 8:**
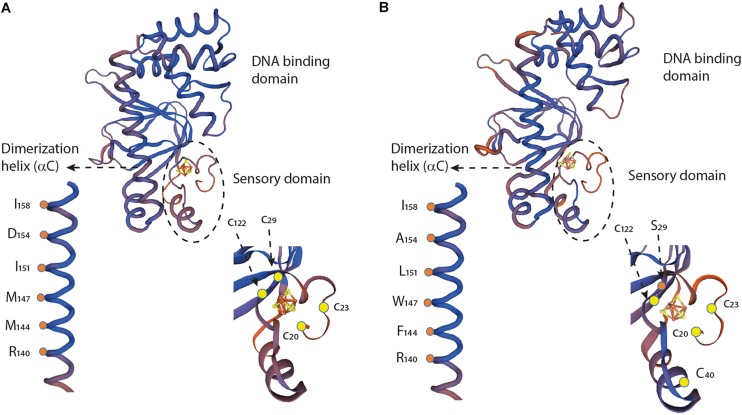
Structural comparison and partial sequence comparison of **(A)** FNR_*EC*_ and **(B)** FNR_AF_. Models of the three-dimensional structures of the respective monomers, showing the sensory domains, dimerization helices, and DNA binding domains, were created using the crystal structure of FNR_*AFi*_ (PDB 5e44) as a template. Cartoons of the sensory domains and dimerization helices are enlarged to illustrate specific amino acid locations discussed in the text. [4Fe-4S]^2+^ centers are illustrated with yellow and red Fe-S cage structures.

Despite the overall similarity of the primary amino acid sequences of FNR_AF_ and FNR_EC_, a number of important differences were observed. It is important to consider how these differences might affect the function of FNR_AF_ and impact how FNR_AF_ coordinates the [4Fe-4S] center and its increased resistance to O_2_. In order to address these issues, an alignment was carried out of the amino acid sequences of FNR_AF_, FNR_EC_, and FNR from *Aliivibrio fisheri* (Figure 7). The primary amino acid sequences were then compared to three dimensional models of FNR_AF_ and FNR_EC_ built using the crystal structure of FNR from *A. fisheri* (FNR_AFi_) as a template [PDB 5CVR (Volbeda et al., 2015)]. In agreement with the amino acid sequence evidence, the model shows that FNR_AF_ shares similar global protein structure with important functional domains of FNR_EC_, displaying a similar spatial distribution with an acceptable QMEAN score of −1.71 (Figure 8). These domains include the sensor domain that comprises a series of structural β-sheets with a [4Fe-4S]^2+^ coordination site, an α-helix promoting protein dimerization, and a DNA-binding domain composed of an HTH motif that allows recognition and binding to transcription factor binding sites (Myers et al., 2013).

#### [4Fe-4S]^2+^ Center Coordination in FNR_AF_

A notable difference in amino acid sequence between FNR_AF_ and FNR_EC_ is in the coordinating ligands of the [4Fe-4S]^2+^ center. In FNR_EC_, coordination is carried out by four cysteines located at positions 20, 23, 29, and 122 (Figure 7). In FNR_AF_, cysteines are conserved at positions 20, 23, and 122 and, based on the 3D model, these are predicted to be located in positions that could potentially allow them to participate in coordinating the [4Fe-4S]^2+^ center (Figure 8). There is no corresponding cysteine at position 29 in FNR_AF_; there is, however, a Ser at this position that could potentially be the fourth coordinating ligand of the [4Fe-4S]^2+^ center. While mutational studies have shown that Ser can serve as a cluster ligand (Fujinaga et al., 1993; Vassiliev et al., 1995; Bentrop et al., 1996; Mansy et al., 2002) naturally occurring serine ligands are rare. Nevertheless, it is worth noting that the LipA enzyme contains an auxiliary [4Fe-4S] cluster that contains a 3Cys/Ser cluster ligation (Harmer et al., 2014).

An alternative hypothesis is that a Cys in FNR_AF_ at position 40 could assume the function of the missing Cys_29_. Although this hypothesis cannot be rejected, we do not favor it. Inspection of the three-dimensional model of FNR_AF_ suggests that the protein is unlikely to be able to fold to bring Cys40 into sufficient proximity to the [4Fe-4S]^2+^ center to facilitate the required coordination.

#### Amino Acid/Structural Changes That Could Help Explain the Observed Stability of FNR_AF_ in Air

One of the major differences of FNR_AF_ compared to FNR_EC_ is the increased stability of the Fe-S cluster in air. Here, we inspect the primary amino acid sequence and postulated 3D structure of FNR_AF_ in order to propose hypotheses for explaining this unusual property.

Amino acid changes around the cluster ligand Cys_23_ have been shown to alter the O_2_ response of FNR in a number of organisms. In FNR_EC_, replacement of Ser_24_, located immediately adjacent to the cluster ligand Cys_23_, by Pro results in increased aerobic FNR activity (Jervis et al., 2009). A natural variant of FNR from *P. denitrificans* has Pro in the position equivalent to Ser_24_ in FNR_EC_ and is at least six times less sensitive to O_2_ than FNR_EC_ (Crack et al., 2016). In *P. putida* a natural variant of FNR has an Arg in position 24 and is more stable to O_2_ than FNR_EC_ (Ibrahim et al., 2015). In *At. ferrooxidans* there is Leu in position 24 (Figure 7) and by analogy, this amino acid substitution could at least partially account for the lower O_2_ reactivity of FNR_AF_.

Amino acid changes at other positions next to the cluster-coordinating Cys residues are also known to influence the aerobic reactivity of FNR_EC_. For example, substitution of Asp_22_ by Ala (Ibrahim et al., 2015) or by Gly (Kiley and Reznikoff, 1991) increased O_2_ activity of FNR. FNR_AF_ has an Asp to His substitution at position 22. Interestingly, it has been found that juxtaposition of His to the cysteine-coordinated [4Fe-3S]^2+^ center of a subgroup of Ni hydrogenases provides stability in the presence of O_2_ (Frielingsdorf et al., 2014; Flanagan et al., 2018). Also, substitution of Leu_28_ by the positively charged His has been shown to stabilize the [4Fe-4S]^2+^ center in FNR_EC_ in the presence of O_2_ (Bates et al., 2000) perhaps by hindering conformational flexibility of the region (Volbeda et al., 2015). FNR_AF_ has the bulky, polar, neutral amino acid Gln in this position and perhaps, like the L_28_F variant of FNR_EC_ (Jervis et al., 2009), this could hinder conformational flexibility by steric hindrance that results in greater activity in O_2_.

Changes in the dimerization helix may also alter the stability of the FNR dimer in O_2_. Two charged residues Arg_140_ and Asp_130_ have been reported to a play key role in the monomer-dimer equilibrium in FNR_EC_ (Moore and Kiley, 2001). An examination of the crystal structure of FNR_AFi_ indicates that these residues could form a salt bridge between the a-C helix (Arg140) of one monomer with the opposite a-B helix (Asp_130_) of the other monomer, perhaps modulating monomer-dimer equilibrium in FNR (Volbeda et al., 2015). Both Arg_140_ and Asp_130_ are conserved in FNR_AF_ implying conservation of the salt bridge and its role in monomer-dimer equilibrium in changing O_2_ environments (Figures 7, 8).

Of particular importance is the observation that when Asp_154_ in the dimer interface of FNR_EC_ is replaced with Ala_154_, FNR_EC_ exhibits increased activity under aerobic conditions (Moore et al., 2006). In FNR_AF_ position 154 is occupied naturally by Ala, strongly suggesting that this change could, at least partially, explain its increased activity in O_2_ (Figures 7, 8).

Hydrophobic interactions have also been shown to be involved in dimer interaction and stabilization. These include Met_144_, Met_147_, Ile_151_, and Ile_158_ that lie on the dimer interface of FNR_EC_ as shown in the three-dimensional model (Figure 8; Moore and Kiley, 2001; Volbeda et al., 2015). Ile_158_ is conserved in FNR_AF_, but the other equivalently positioned residues in FNR_AF_ are Phe_144_, Trp_147_, and Leu_151_, respectively. All are hydrophobic and potentially play a role in dimer stabilization. Of interest is the possibility that in FNR_AF_ Trp_147_ of one monomer helix and Phe_144_ of the complementary monomer helix could interact through stacking of their respective aromatic rings, potentially providing additional stability to the interacting helices as has been observed in other proteins (McGaughey et al., 1998; Budyak et al., 2013; Madhusudan Makwana and Mahalakshmi, 2015).

Another difference is the presence of a truncated and divergent N terminal region of FNR_AF_ compared to FNR_EC,_ in which FNR_AF_ has only 7 amino acids just prior to the first Cys involved in [4Fe-4S]^2+^ center coordination instead of the 19 observed in FNR_EC_ (Figure 7). These amino acids form part of a flexible region with no predicted secondary structure. The truncation in FNR_AF_ does not appear to be a result of sequence mis-annotation e.g., incorrect translation start site. Interestingly, deletion of N-terminal amino acid residues 2 to 16 and 2 to 17 FNR_EC_, increased FNR activity under aerobic conditions (Yan and Kiley, 2008). These results suggest that the N-terminal region also contributes to the lability of the [4Fe-4S] cluster of FNR to O_2_ and that the removal of amino acids in this region may act to increase the stability of the cluster to O_2_. How these changes operate is not known.

## Additional Discussion

Anaerobic culturing of *At. ferrooxidans* confirmed previous reports that it is capable of growth using Fe^3+^ as the final electron acceptor (Ohmura et al., 2002; Osorio et al., 2013) and that anaerobic growth is slower than with O_2_ as the electron acceptor (Osorio et al., 2013; Figure 1). The slower rate of growth for anaerobic cultures may be due to the greater amount of energy available from sulfur oxidation using O_2_ as terminal electron acceptor (−124 kcal/atom S^0^) compared with Fe^3+^ reduction (−75 Kcal/atom S^0^).

The ability to transition from aerobic reduction of O_2_ to utilizing ferric iron as a terminal electron acceptor suggests that *At. ferrooxidans* must regulate the expression of alternative electron transfer chains used in energy conservation. In this study, we provide evidence that *At. ferrooxidans*, a model organism for studying life at extremely low pH, contains a FNR-like protein (FNR_AF_) that is a member of the CRP FNR superfamily of regulators. FNR_AF_ exhibits sequence (Figure 2) and structural similarity (Figure 8) with the archetypal FNR from *E. coli* (FNR_EC_) that was recently deduced from the crystal structure of FNR from *Al. fisheri* (Mettert and Kiley, 2018). FNR_AF_ reacts with antibodies prepared against FNR_EC_ (Figure 5A) and is able to drive expression from the FNR-responsive *E. coli* P*narG* promoter, suggesting that it is functionally active as an FNR-like protein at least in the surrogate host *E. coli*.

Despite high levels of structural and protein sequence similarity, FNR_AF_ exhibits several properties that differ from FNR_EC_. First, RNA encoded by *fnr*_AF_, although detected in aerobic cultures, increases in amount in the stationary phase of anaerobically grown cultures (Figure 4), suggesting that depletion of O_2_, and/or culture age upregulate *fnr* expression or modifies post-transcriptional processing of *fnr* RNA. Upregulation of *fnr* in anaerobic conditions has also been observed in *B. subtilis* (Cruz Ramos et al., 1995). In contrast, although there is negative auto-regulation of FNR_EC_ expression (Mettert and Kiley, 2007), in *E. coli* it has been demonstrated that FNR activity is predominantly regulated at the protein level where FNR appears to cycle between active [4Fe-4S]^2+^, inactive [2Fe-2S], and apo forms, with the level of O_2_ determining which form predominates and therefore, the extent to which FNR is transcriptionally active. Such a mechanism requires that the levels of FNR in the cell are tightly controlled (Spiro and Guest, 1987; Sutton et al., 2004; Mettert and Kiley, 2007; Jervis et al., 2009). The suggestion that expression of *fnr*_AF_ is regulated opens up opportunities to investigate the underlying mechanism(s) involved.

Another important consideration is the significantly increased stability of FNR_AF_ compared to FNR_EC_. What could be the evolutionary advantage of this strategy? We hypothesize that it allows the control of genes in its network over a wide range of O_2_ concentrations without the need to resort to recycling between active and inactive forms of FNR, as in *E. coli*, or to differential transcriptional regulation of FNR as exhibited by *B. subtilis*, *P. putida, H. seropedicae*, *B. cenocepacia*, and *R. eutropha*. These mechanisms are energetically costly and time consuming. Speed of response to increased environmental concentrations of O_2_ might be particularly critical for *At. ferrooxidans* as it needs to transition rapidly from anaerobic to highly oxidizing Fe-rich environments at very low pH such as found in bioheaps for industrial copper recovery (Jerez, 2008) and in biofilms in naturally occurring acidic environments (Wilmes et al., 2009; Liljeqvist et al., 2015) and these responses need to be made in an organism with a relatively slow growth rate and whose energy budget allocation is restricted by living at the thermodynamic edge of life.

## Data Availability

Publicly available datasets were analyzed in this study. This data can be found here: https://www.ncbi.nlm.nih.gov/genome/1014?genome_assembly_id=300479.

## Author Contributions

DH, EJ, and PK designed the study. HO and EM carried out the experiments. All authors analyzed the data. DH and MD drafted the manuscript and all authors agreed on the final version.

## Conflict of Interest Statement

The authors declare that the research was conducted in the absence of any commercial or financial relationships that could be construed as a potential conflict of interest.

## Footnotes

1 http://www.graphpad.com/scientific-software/prism/

2 www.uniprot.org/uniprot/P0A9E5
